# Supplementary material for: Dietary restriction transforms the mammalian protein persulfidome in a tissue-specific and cystathionine γ-lyase-dependent manner
Source: Nat Commun. 2021 Mar 19;12:1745. doi: 10.1038/s41467-021-22001-w (PMC7979915; doi:10.1038/s41467-021-22001-w)
Supplement: Supplementary file 1 — Supplementary Information [file 41467_2021_22001_MOESM1_ESM.pdf]

Supplementary Information document for:

Dietary restriction transforms the mammalian protein persulfidome in a tissue-specific and cystathionine γ-lyase-dependent manner

Authors: Nazmin Bithi[1], Christopher Link[1], Yoko O. Henderson[1], Suzie Kim[1], Jie Yang[1], Ling Li[2], Rui Wang[3], Belinda Willard[2], and Christopher Hine[1*]

Affiliations:

[1]Department of Cardiovascular and Metabolic Sciences, Cleveland Clinic Lerner Research Institute, Cleveland, OH 44195, USA.

[2]Proteomics and Metabolomics Core Laboratory, Cleveland Clinic Lerner Research Institute, Cleveland, OH 44195, USA

[3]Department of Biology, Faculty of Science, York University, Toronto, Canada M3J 1P3.

*To whom correspondence should be addressed: Christopher Hine, email: hinec@ccf.org

In this Supplementary Information document, the following is provided:

1) Supplementary Figures and their Legends: (1-9)

Supplementary Figure 1: Food intake and changes in body mass as a result of 1 week 50% dietary restriction.
Supplementary Figure 2: Modified biotin thiol assay (BTA) to isolate and detect persulfidated proteins in tissues.
Supplementary Figure 3: Pathway enrichment for persulfidated proteins not significantly changed by diet.
Supplementary Figure 4: Persulfidation analysis and pathway enrichment in heart and plasma.
Supplementary Figure 5: Persulfidation analysis in tissues from aged mice on long-term ad libitum versus Every Other Day (EOD) fasting diets
Supplementary Figure 6: NaHS pretreatment augments H2S production capacity of CGL
Supplementary Figure 7: Persulfidation analysis in CGL KO liver and kidney.
Supplementary Figure 8: Persulfidation analysis in CGL KO muscle, brain, and heart.
Supplementary Figure 9: Orthogonal protein and peptide based Biotin Thiol Assay (BTA) approaches to detect CGL dependence for DR induced persulfidation shifts in kidney and brain

2) Supplementary References

1) Supplementary Figures

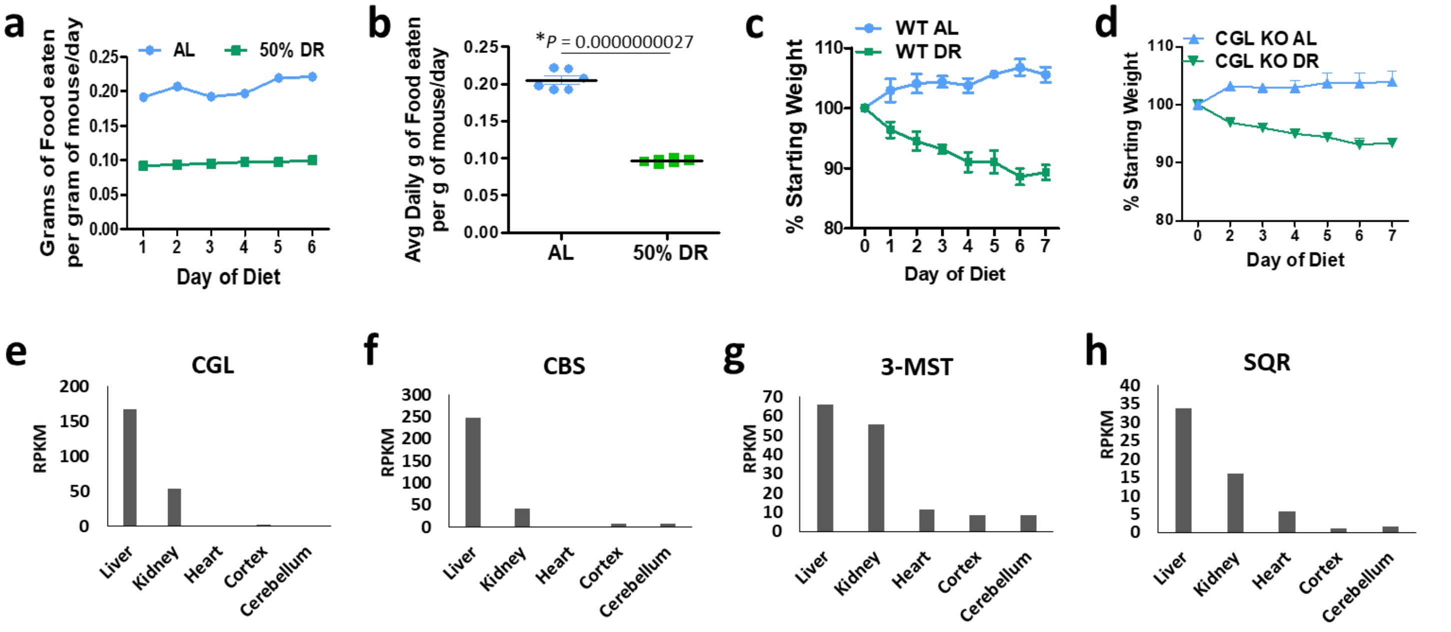

Supplementary Figure 1: **Food intake and changes in body mass as a result of 1 week 50% dietary restriction.** (**a-b**) Daily (**a**) and average (**b**) food intake of modified AIN-93G rodent diet displayed as grams of food eaten per gram of mouse body mass per day over a 1 week period of *ad libitum* (AL) (*n*=7 mice/group) or 50% dietary restriction (DR) (*n*=8 mice/group) feeding. Asterisk indicates the statistical significance between AL versus DR with the *P* value given from a two-sided Student's *t* test. Means are shown with error bars ± SEM. (**c-d**) Body masses of cystathionine γ-lyase (CGL) wildtype (WT) (**c**) and total body CGL knock out (KO) (**d**) mice over the 7-day dietary intervention normalized to % initial starting weight. WT AL *n*=4 mice/group, WT DR *n*=5 mice/group, KO AL *n*=3 mice/group, and KO DR *n*=3 mice/group. Means are shown with error bars ± SEM. (**e-h**) RNA expression profiling data sets of H_2S producing (**e-g**) and consuming (**h**) proteins generated by the Mouse ENCODE project [1] and extracted from the NCBI Mouse Gene Database at the following webpages: CGL: [https://www.ncbi.nlm.nih.gov/gene/107869]; CBS (cystathionine β-synthase) [https://www.ncbi.nlm.nih.gov/gene/12411]; 3-MST (3-mercaptopyruvate sulfurtransferase) [https://www.ncbi.nlm.nih.gov/gene/246221]; SQR (sulfide:quinone oxidoreductase) [https://www.ncbi.nlm.nih.gov/gene/59010]. RPKM = reads per kilobase of transcript, per million mapped reads. Source data are provided as a Source Data file. See also Figure 1.

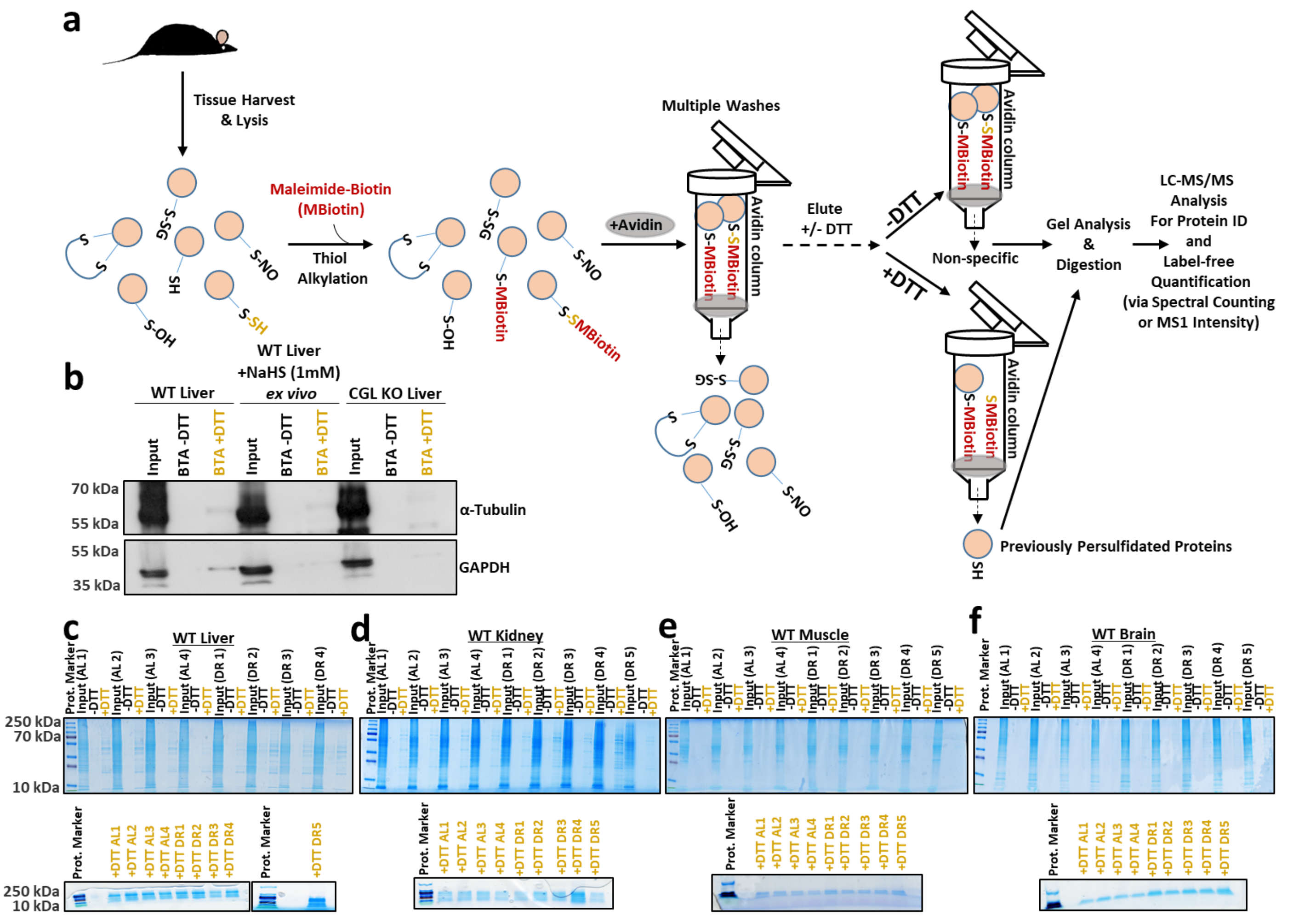

Supplementary Figure 2: **Modified biotin thiol assay (BTA) to isolate and detect persulfidated proteins in tissues**. (**a**) Schematic of the adapted biotin-thiol-assay (BTA) to isolate, detect, and quantify persulfidated proteins in mouse tissue. Tissues were homogenized and lysed, the protein concentrations equally normalized, and then lysates subjected to alkylation and binding to maleimide-biotin (MBiotin). Subsequent addition of biotin bound and unbound proteins into an avidin column isolated those bound to biotin, with several buffered wash steps and elution without dithiothreitol (-DTT) to remove non-specific non-persulfidated proteins attached to the column. A final buffered elution with DTT (+DTT) reduces and cleaves the disulfide bond between the cysteine-attached sulfur and the persulfide (shown in yellow text) bound to the biotin/avidin column, thus eluting the previously persulfidated proteins for downstream gel-based analysis (**b-f**) as well as mass spectrometry based LC-MS/MS analysis coupled with label-free spectral counting or MS1 intensity to identify and measure relative persulfidated protein abundance as a function of diet and/or genotype. (**b**) Validation of the BTA via Western blot analysis of liver lysates treated *ex vivo* -/+ 1 mM sodium hydrosulfide (NaHS) from CGL WT and KO mice for the known persulfidated proteins α-tubulin and GAPDH following application of the BTA. (**c-f**) SDS-PAGE gel electrophoresis followed by colloidal staining on liver (**c**), kidney (**d**), muscle (**e**), and brain (**f**) protein lysate input loading controls as well as –DTT and +DTT eluates from the BTA derived from *ad libitum* (AL, *n*=4) or 50% dietary restriction (DR; *n*=5) fed cystathionine γ-lyase (CGL) WT mice (top gels). Bottom SDS-PAGE gel images are from the same +DTT eluates shown in the upper gels but run for a short period of time; 13-15 minutes, prior to excising the entire protein lane to ensure all of that tissue's persulfidated proteins across the full spectrum of protein masses are accounted for in the subsequent downstream LC-MS/MS proteomics analysis. Yellow font for +DTT indicates the experimental conditions in which the persulfidated proteins were eluted and isolated for future gel and LC-MS/MS proteomics analysis. Source data are provided as a Source Data file. See also Figure 2.

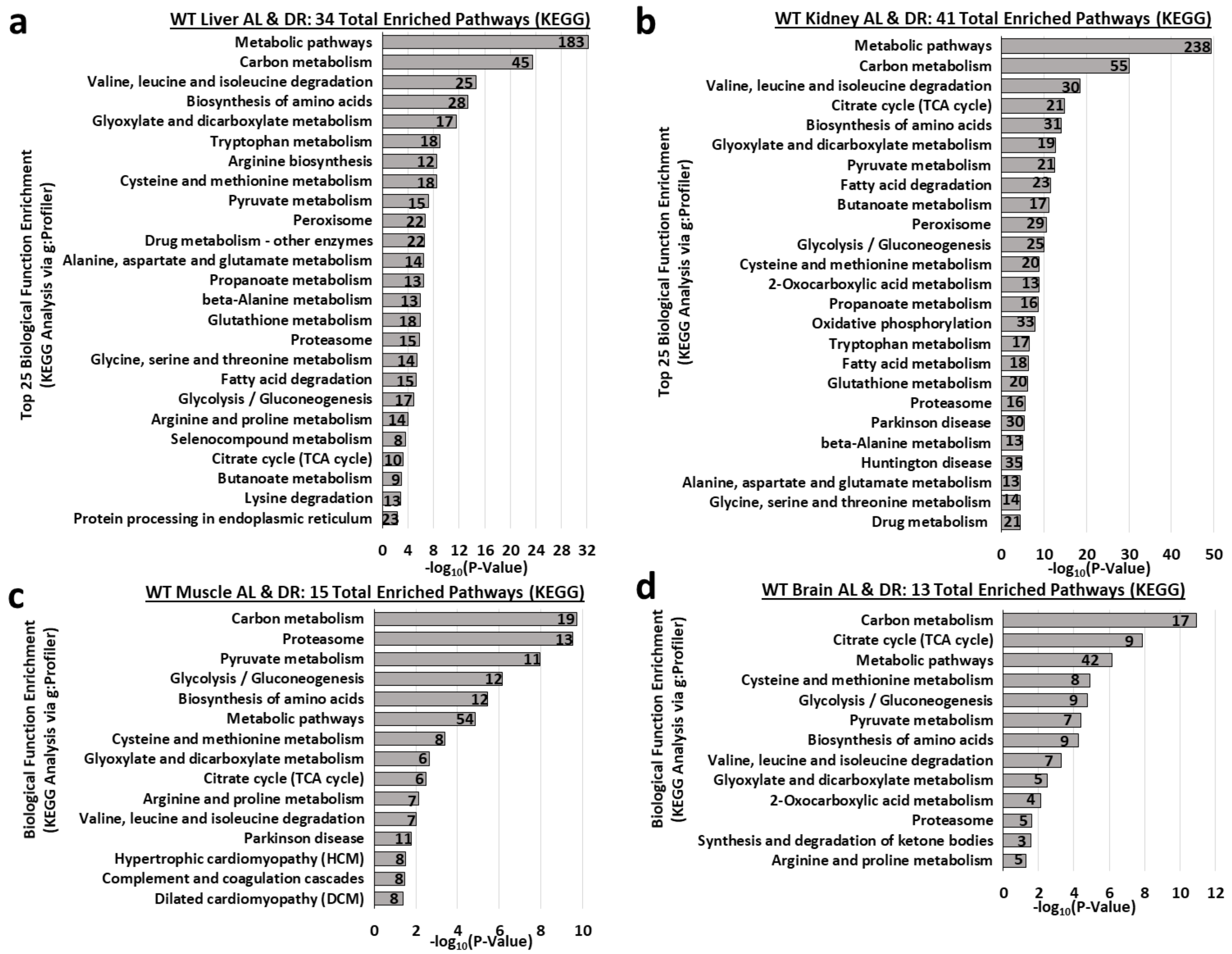

Supplementary Figure 3: **Biological function and pathway enrichment for persulfidated proteins not significantly changed by diet.** (**a-d**) KEGG biological function and pathway enrichment via g:Profiler analysis of persulfidated proteins whose relative abundance was not biologically and/or significantly changed under AL or DR feeding in liver (**a**), kidney (**b**), muscle (**c**), and brain (**d**). The numbers within the bars indicate individual persulfidated proteins identified for that specific pathway. *n* = 9 mice in total; 4 per WT AL group and 5 per WT DR group. Statistical significance for pathway enrichment plotted as the adjusted $-\log_{10}$ (*P*-Value) and were auto-calculated via the g:Profiler g:SCS algorithm for KEGG database that utilized multiple testing correction. See also Figure 3.

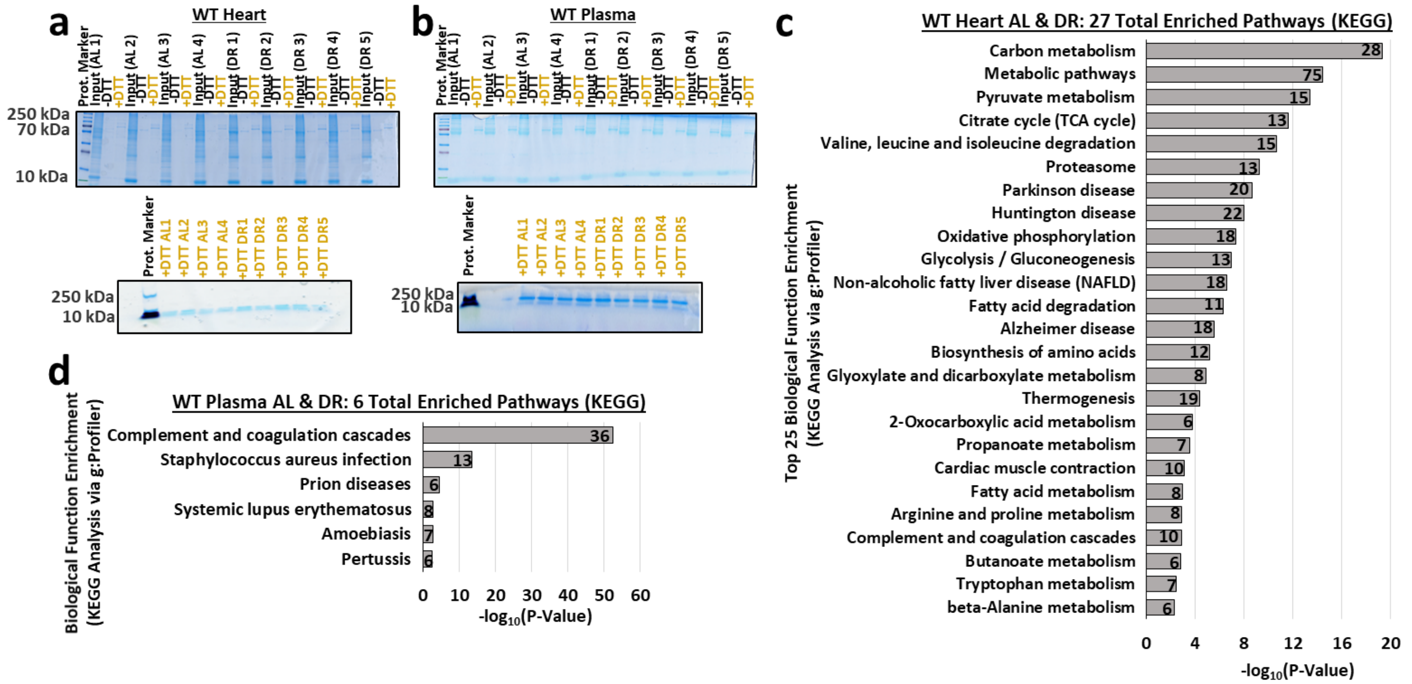

Supplementary Figure 4: **Persulfidation analysis and pathway enrichment in heart and plasma**. (**a-b**). SDS-PAGE gel electrophoresis followed by colloidal staining on heart (**a**), and plasma (**b**) protein lysate input loading controls as well as –DTT and +DTT eluates from the BTA derived from AL (*n*=4) or DR (*n*=5) fed cystathionine γ-lyase (CGL) WT mice (top gels). Bottom SDS-PAGE gel images are from the same +DTT eluates shown in the upper gels but run briefly prior to excising the entire protein lane to ensure all of that tissue's persulfidated proteins across the full spectrum of masses are accounted for in the subsequent downstream LC-MS/MS proteomics analysis. Yellow font for +DTT indicates the experimental conditions in which the persulfidated proteins were eluted and isolated for future gel and LC-MS/MS proteomics analysis. (**c-d**) KEGG biological function and pathway enrichment via g:Profiler analysis of persulfidated proteins whose relative abundance was not changed under AL or DR feeding in heart (**c**), and plasma (**d**). The numbers inside the bars indicate the persulfidated proteins involved in that specific pathway. n = 9 mice in total; 4 in the AL group and 5 in the DR group. Statistical significance for pathway enrichment plotted as the adjusted $-\log_{10}$ (P-Value) and were auto-calculated via the g:Profiler g:SCS algorithm for KEGG database that utilized multiple testing correction. Source data are provided as a Source Data file. See also Figure 4.

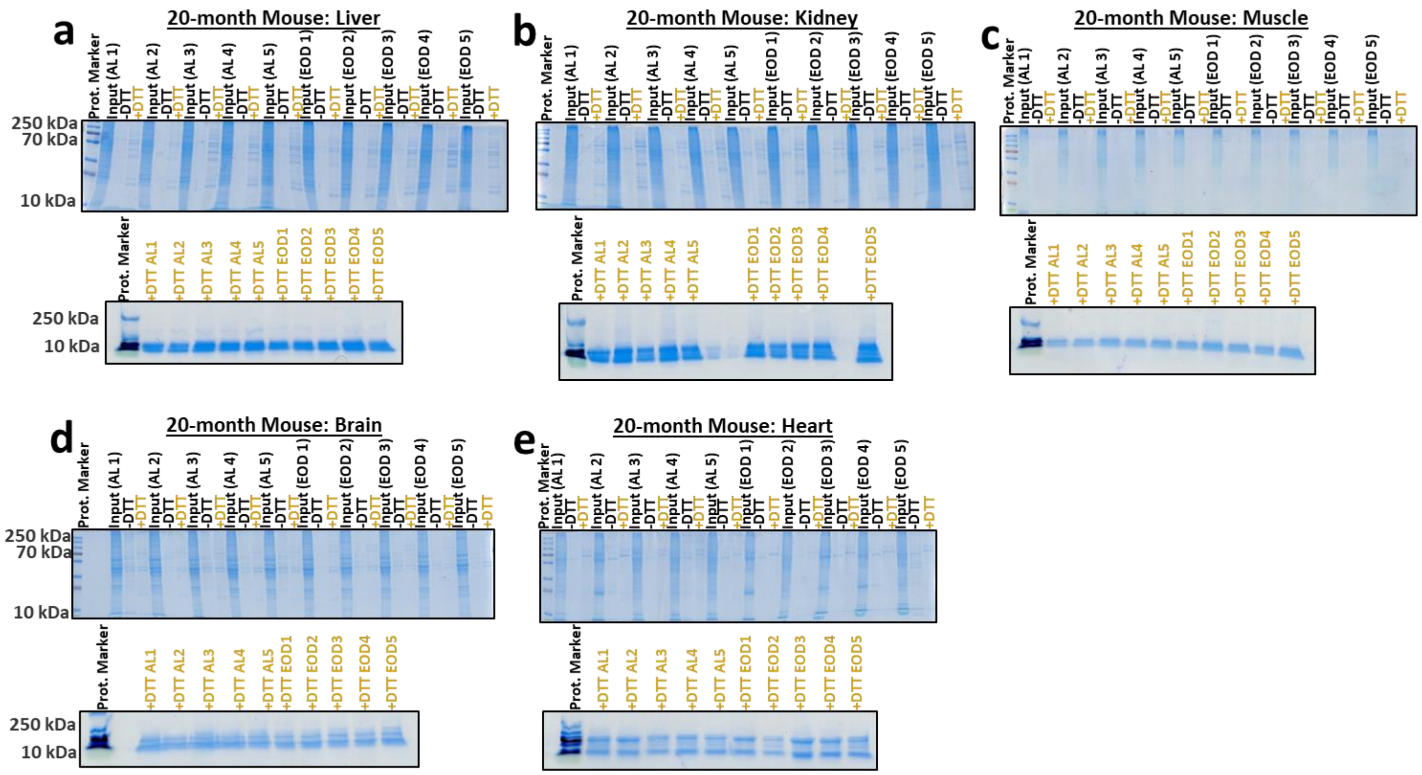

Supplementary Figure 5: **Persulfidation analysis in tissues from aged mice on long-term *ad libitum* versus Every Other Day (EOD) fasting diets**. (**a-e**). SDS-PAGE gel electrophoresis followed by colloidal staining on liver (**a**), kidney (**b**), muscle (**c**), brain (**d**), and heart (**e**) lysate input loading controls as well as –DTT and +DTT eluates from the BTA derived from AL fed (*n*=5) or 2.5 months of EOD fasting (*n*=5) 20-month old C57BL/6 male mice (top gels). Bottom SDS-PAGE gel images are from the same +DTT eluates shown in the upper gels but run briefly prior to excising the entire protein lane to ensure all of that tissue's persulfidated proteins across the full spectrum of masses are accounted for in the subsequent downstream LC-MS/MS proteomics analysis. Yellow font for +DTT indicates the experimental conditions in which the persulfidated proteins were eluted and isolated for future gel and LC-MS/MS proteomics analysis. Source data are provided as a Source Data file. See also Figure 5.

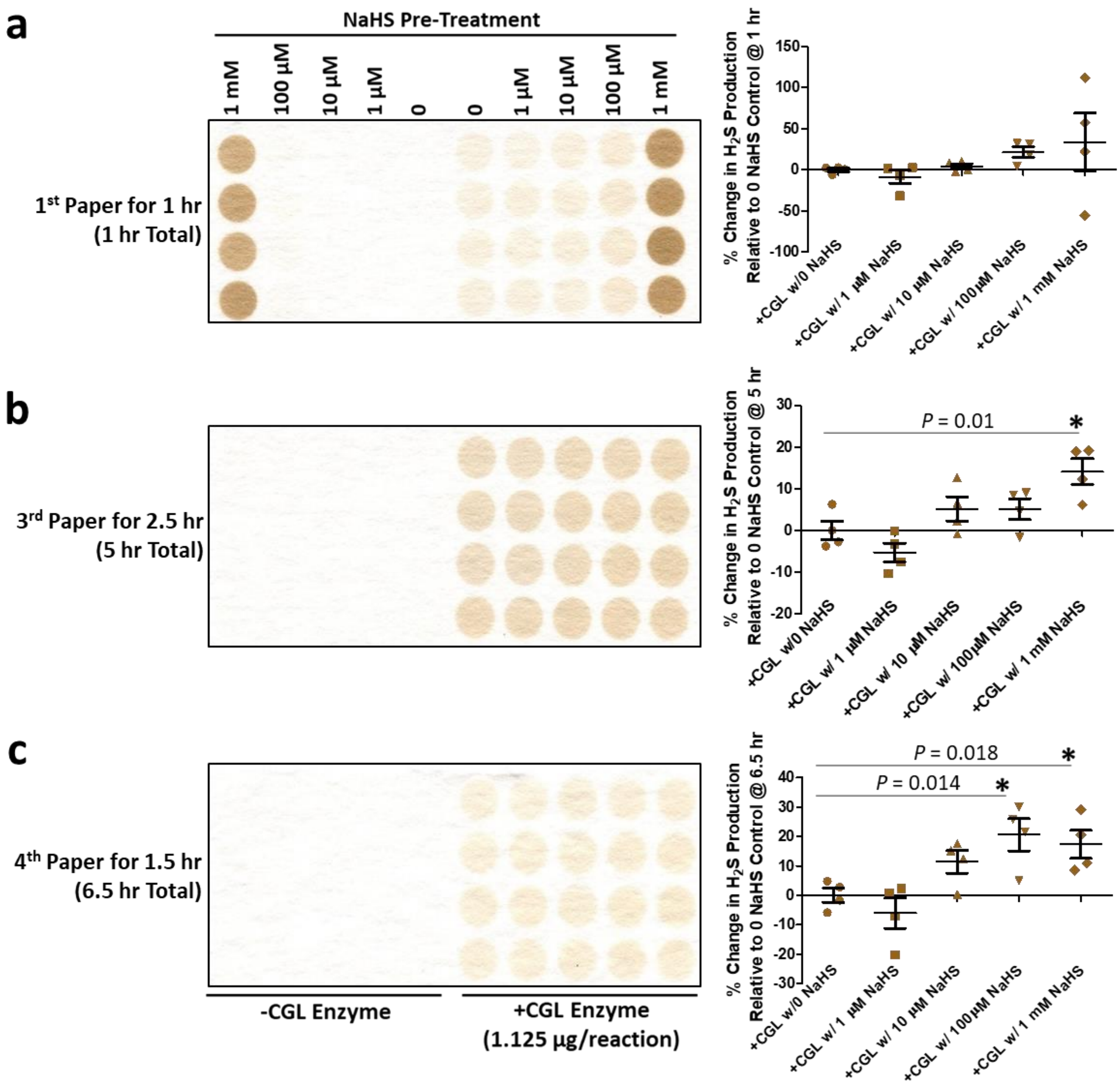

Supplementary Figure 6: NaHS pretreatment augments H_2S production capacity of CGL. (**a-c**) Endpoint detection via the lead acetate/lead sulfide method and quantification via integrated densitometry of H_2S production from purified CGL enzyme in L-cysteine and pyridoxal phosphate containing reaction mixes with various doses of NaHS after indicated incubation periods at 37°C. New lead acetate detection papers were placed on the reaction plates at the start of each incubation period, and the (-)CGL lead sulfide integrated density values were subtracted from their dose-corresponding (+)CGL lead sulfide integrated density values prior to plotting the % change in CGL-derived H2S production compared to the (+)CGL w/ 0 NaHS control. Error bars are ± SEM. The given individual *P*-values are calculated from two-sided *t*-test without multiple comparison test, while asterisks indicate $P < 0.05$ calculated from 1-way ANOVA with Dunnet's Multiple Comparison Test. n = 4/reaction condition. Source data are provided as a Source Data file. See also Figure 6.

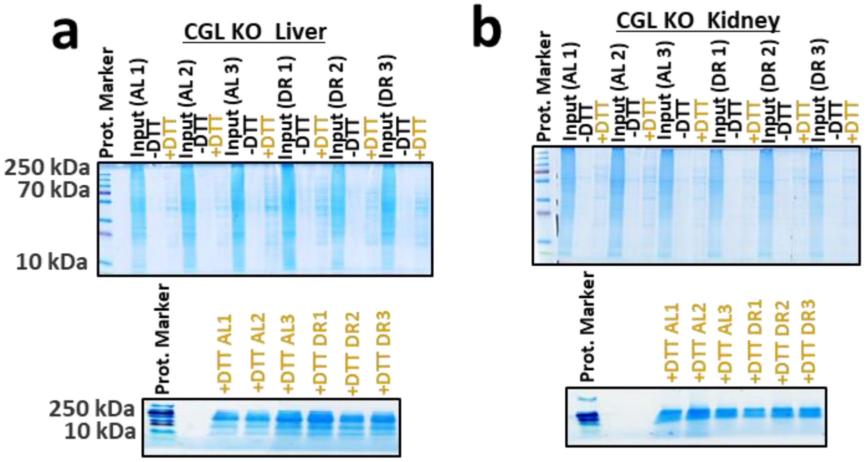

Supplementary Figure 7: **Persulfidation analysis in CGL KO liver and kidney. (a-b)** SDS-PAGE gel electrophoresis followed by colloidal staining on liver (**a**) or kidney (**b**) protein lysate input loading controls as well as –DTT and +DTT eluates from the BTA derived from KO AL (*n*=3) or KO DR (*n*=3) fed 6-months old cystathionine γ-lyase (CGL) KO mice (top gels). Bottom SDS-PAGE gel images are from the same +DTT eluates shown in the upper gels but run briefly prior to excising the entire protein lane to ensure all of the persulfidated proteins across the full spectrum of masses are accounted for in the subsequent downstream LC-MS/MS proteomics analysis. Yellow font for +DTT indicates the experimental conditions in which the persulfidated proteins were eluted and isolated for future gel and LC-MS/MS proteomics analysis. Source data are provided as a Source Data file. See also Figure 7.

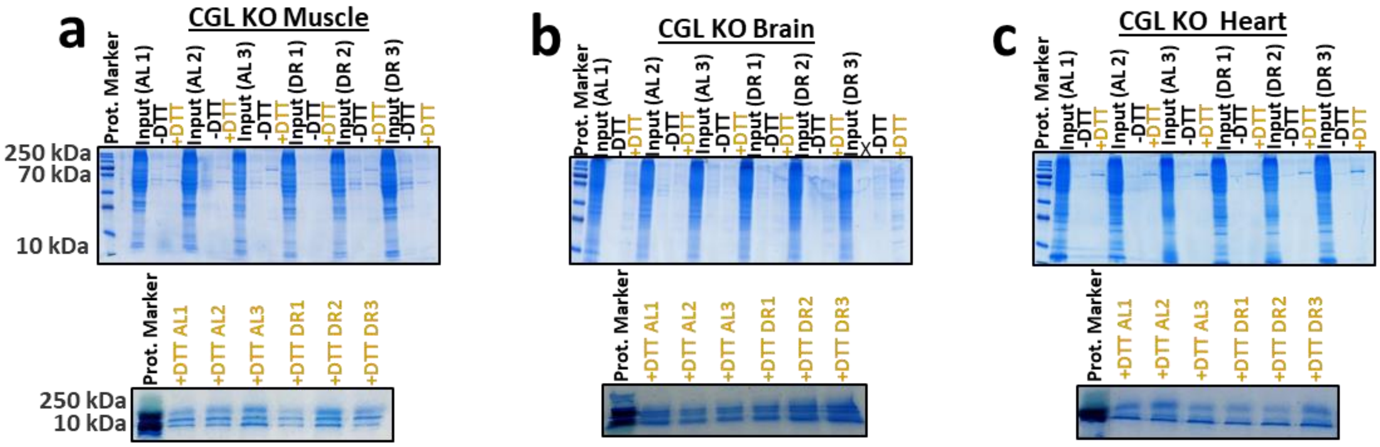

Supplementary Figure 8: Isolation and detection of persulfidated proteins in muscle, brain, and heart of CGL KO mice. (a-c) SDS-PAGE gel electrophoresis followed by colloidal staining on muscle (**a**), brain (**b**) and heart (**c**) protein lysate input loading controls as well as –DTT and +DTT eluates from the BTA derived from KO AL (*n*=3) or KO DR (*n*=3) fed cystathionine γ-lyase (CGL) KO mice (top gels). Bottom SDS-PAGE gel images are from the same +DTT eluates shown in the upper gels but run briefly prior to excising the entire protein lane to ensure all of the persulfidated proteins across the full spectrum of masses are accounted for in the subsequent downstream LC-MS/MS proteomics analysis. Yellow font for +DTT indicates the experimental conditions in which the persulfidated proteins were eluted and isolated for future gel and LC-MS/MS proteomics analysis. Source data are provided as a Source Data file. See also Figure 8.

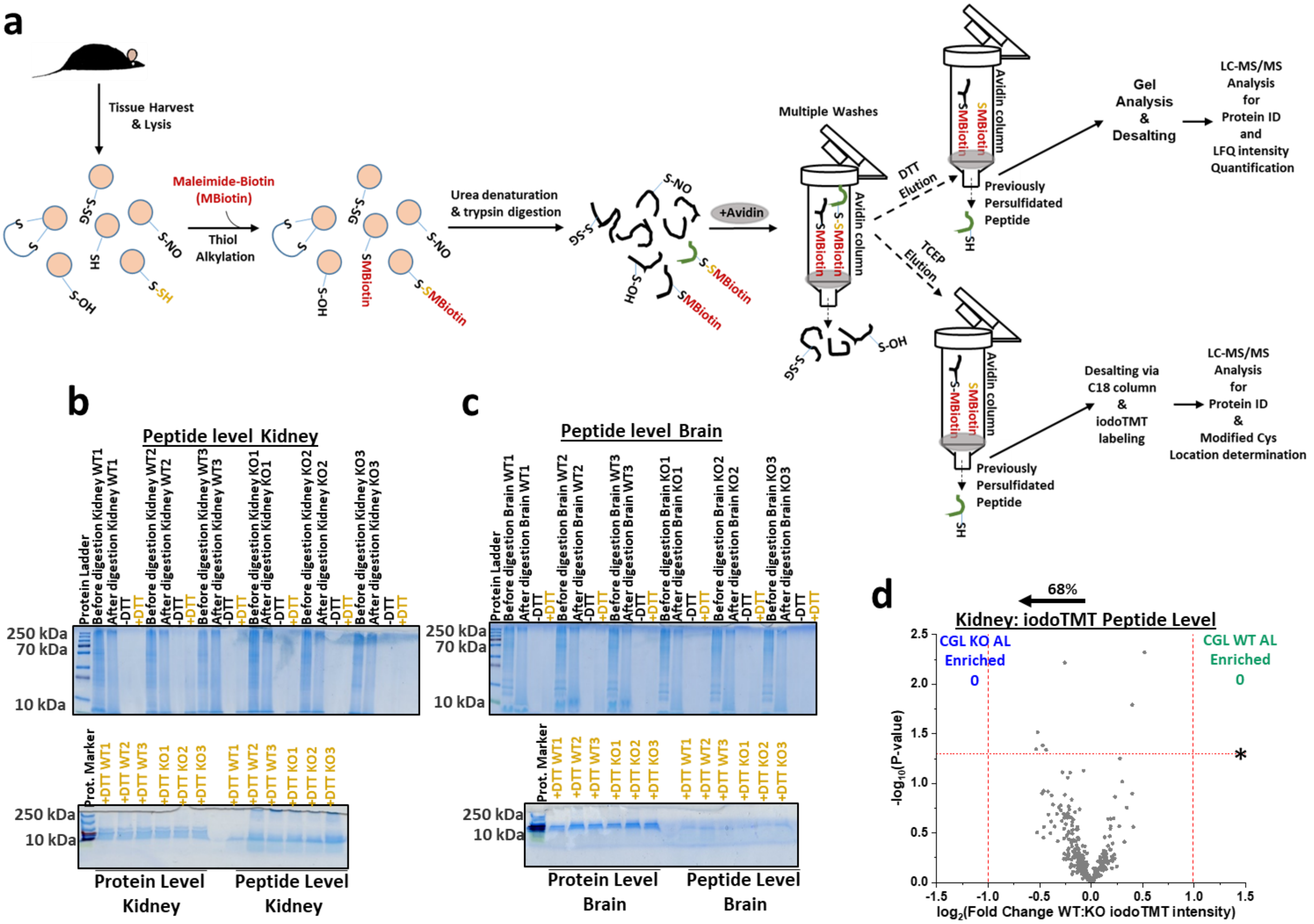

Supplementary Figure 9: Orthogonal protein- and peptide-based Biotin Thiol Assay (BTA) approaches to detect CGL-dependence for DR-induced persulfidation shifts in kidney and brain. (**a**) Schematic of the unlabeled and iodoTMT labeled peptide based BTA. Kidney and brain extracts were subjected to alkylation and binding to maleimide-biotin (MBiotin). Subsequently, samples were denatured with urea and digested with trypsin, passed through an avidin column, washed several times and then persulfidated peptides eluted with dithiothreitol (-DTT) for unlabeled analysis or TCEP (tris(2-carboxyethyl)phosphine) for labeled analysis. Unlabeled proteins and peptides underwent in-gel electrophoresis and digestion (see b-c). Peptides for labeled analysis were desalted via C18 column, free thiols alkylated with iodoTMT 6plex tags, and samples from individual mice combined. Both unlabeled and iodoTMT labeled peptides were analyzed by LC-MS/MS. **(b-c)** SDS-PAGE gel electrophoresis followed by colloidal staining for input controls and post-BTA +/- DTT eluates from kidney (**b**) and brain (**c**) derived from 1-year old CGL WT and KO mice (n = 3 mice/group). Input controls show pre- and post-trypsin digestion (top gels). The bottom SDS-PAGE gel are from protein and peptide level +DTT eluates, run briefly prior to excising entire bands to ensure capture of proteins/peptides across the full spectrum of masses for downstream LC-MS/MS analysis. Yellow font for +DTT indicates the experimental conditions in which the persulfidated proteins were eluted for gel and LC-MS/MS proteomics analysis. **(d)** Volcano plot showing intensity shifts in kidney persulfidated proteins derived from iodoTMT labeled peptide level BTA from *ad libitum* fed CGL WT and KO mice (n = 3mice/group). The $\log_2$(Fold Change WT:KO) X-axis displays the average fold change in intensity for each protein, and the $-\log_{10}$ Y-axis displays the calculated P-value from a two-sided Student's t test when comparing the individual intensity values for each protein from WT versus KO mice. The non-axial red dotted vertical lines highlight the biological significance threshold of +/-2-fold change in intensity, while the non-axial red dotted horizontal line with asterisk highlights the statistical significance threshold of P <0.05. The percentage and direction of proteins skewed toward CGL KO is provided above the tissue label. Source data are provided as a Source Data file. See also Figure 9.

2) Supplementary References

1 Yue, F. *et al.* A comparative encyclopedia of DNA elements in the mouse genome. *Nature* **515**, 355-364, doi:10.1038/nature13992 (2014).
