# Supplementary material for: Dietary restriction transforms the mammalian protein persulfidome in a tissue-specific and cystathionine γ-lyase-dependent manner
Source: Nat Commun. 2021 Mar 19;12:1745. doi: 10.1038/s41467-021-22001-w (PMC7979915; doi:10.1038/s41467-021-22001-w)
Supplement: Supplementary file 3 — Description of Additional Supplementary Files [file 41467_2021_22001_MOESM3_ESM.docx]

**Descriptions of Additional Supplementary Files**

**A)** File Name: **Supplemental Information**

Description: The Supplementary Information file contains the Supplementary Figures and their Legends (1-9), and Supplementary References as described below:

i) Supplementary Figures 1-9

Supplementary Figure 1: Food intake and changes in body mass as a result of 1 week 50% dietary restriction.

Supplementary Figure 2: Modified biotin thiol assay (BTA) to isolate and detect persulfidated proteins in tissues.

Supplementary Figure 3: Pathway enrichment for persulfidated proteins not significantly changed by diet.

Supplementary Figure 4: Persulfidation analysis and pathway enrichment in heart and plasma.

Supplementary Figure 5: Persulfidation analysis in tissues from aged mice on long-term ad libitum versus Every Other Day fasting diets

Supplementary Figure 6: NaHS pretreatment augments H_2_S production capacity of CGL

Supplementary Figure 7: Persulfidation analysis in CGL KO liver and kidney.

Supplementary Figure 8: Persulfidation analysis in CGL KO muscle, brain, and heart.

Supplementary Figure 9: Orthogonal protein and peptide based Biotin Thiol Assay (BTA) approaches to detect CGL dependence for DR induced persulfidation shifts in kidney and brain

ii) Supplementary References

**B)** File Name: **Source Data**

Description: **Source images and data for dot plots/graphs.** Uncropped images of gels and lead acetate/lead sulfide papers and/or numerical data used in the dot plots/graphs in the main and supplementary figures. Each tab contains the images and/or data for the noted figure/supplementary figure. Included is material for Figure 1, Supplementary Figure 1, Supplementary Figure 2, Supplementary figure 4, Supplementary Figure 5, Figure 6, Supplementary Figure 6, Supplementary Figure 7, Figure 8, Supplementary Figure 8, and Supplementary Figure 9.

**C)** **Supplementary Data files 1-39**

Description: Each individual data file (1-39) contains mass spectrometry and proteomics-related data.

File Name: **Supplementary Data 1_WT LIVER Persulfidome**

Description: **6-month old CGL WT liver persulfidome.** Information on the tissue-specific persulfidome, including protein names, accession numbers, and the DR to AL ratio from spectral counting (tab 1) or MS1 intensity (tab 2). The *P*-value is calculated by comparing the individual spectral count or intensity values for each identified persulfidated protein in a specific tissue from AL versus DR fed mice via a 2-sided Student’s *t* test. AL; *n* = 4 mice/group versus DR; *n* = 5 mice/group. The accompanying volcano plot displays the differentially abundant persulfidated proteins. The log_2_(Fold Change DR:AL) X-axis displays the average fold change in spectral counts or intensity values for each identified persulfidated protein while the –log_10_ Y-axis displays the calculated *P*-value. The non-axial red dotted vertical lines highlight the biological significance threshold of +/-2-fold change in spectral counts or intensity between DR versus AL, while the non-axial red dotted horizontal line with asterisk highlights the statistical significance threshold of *P* <0.05. Blue (AL enriched) and green (DR enriched) colored rows and dots indicate persulfidated proteins reaching both biological- and statistical- thresholds. Gray color rows and dots indicate persulfidated proteins not reaching the criteria for both biological and statistical significance under either diet.

File Name: **Supplementary Data 2_WT KIDNEY Persulfidome**

Description: **6-month old CGL WT kidney persulfidome.** Information on the tissue-specific persulfidome, including protein names, accession numbers, and the DR to AL ratio from spectral counting (tab 1) or MS1 intensity (tab 2). The *P*-value is calculated by comparing the individual spectral count or intensity values for each identified persulfidated protein in a specific tissue from AL versus DR fed mice via a 2-sided Student’s *t* test. AL; *n* = 4 mice/group versus DR; *n* = 5 mice/group. The accompanying volcano plot displays the differentially abundant persulfidated proteins. The log_2_(Fold Change DR:AL) X-axis displays the average fold change in spectral counts or intensity values for each identified persulfidated protein while the –log_10_ Y-axis displays the calculated *P*-value. The non-axial red dotted vertical lines highlight the biological significance threshold of +/-2-fold change in spectral counts or intensity between DR versus AL, while the non-axial red dotted horizontal line with asterisk highlights the statistical significance threshold of *P* <0.05. Blue (AL enriched) and green (DR enriched) colored rows and dots indicate persulfidated proteins reaching both biological- and statistical- thresholds. Gray color rows and dots indicate persulfidated proteins not reaching the criteria for both biological and statistical significance under either diet.

File Name: **Supplementary Data 3_WT MUSCLE Persulfidome**

Description: **6-month old CGL WT muscle persulfidome.** Information on the tissue-specific persulfidome, including protein names, accession numbers, and the DR to AL ratio from spectral counting (tab 1) or MS1 intensity (tab 2). The *P*-value is calculated by comparing the individual spectral count or intensity values for each identified persulfidated protein in a specific tissue from AL versus DR fed mice via a 2-sided Student’s *t* test. AL; *n* = 4 mice/group versus DR; *n* = 5 mice/group. The accompanying volcano plot displays the differentially abundant persulfidated proteins. The log_2_(Fold Change DR:AL) X-axis displays the average fold change in spectral counts or intensity values for each identified persulfidated protein while the –log_10_ Y-axis displays the calculated *P*-value. The non-axial red dotted vertical lines highlight the biological significance threshold of +/-2-fold change in spectral counts or intensity between DR versus AL, while the non-axial red dotted horizontal line with asterisk highlights the statistical significance threshold of *P* <0.05. Blue (AL enriched) and green (DR enriched) colored rows and dots indicate persulfidated proteins reaching both biological- and statistical- thresholds. Gray color rows and dots indicate persulfidated proteins not reaching the criteria for both biological and statistical significance under either diet.

File Name: **Supplementary Data 4_WT BRAIN Persulfidome**

Description: **6-month old CGL WT brain persulfidome.** Information on the tissue-specific persulfidome, including protein names, accession numbers, and the DR to AL ratio from spectral counting (tab 1) or MS1 intensity (tab 2). The *P*-value is calculated by comparing the individual spectral count or intensity values for each identified persulfidated protein in a specific tissue from AL versus DR fed mice via a 2-sided Student’s *t* test. AL; *n* = 4 mice/group versus DR; *n* = 5 mice/group. The accompanying volcano plot displays the differentially abundant persulfidated proteins. The log_2_(Fold Change DR:AL) X-axis displays the average fold change in spectral counts or intensity values for each identified persulfidated protein while the –log_10_ Y-axis displays the calculated *P*-value. The non-axial red dotted vertical lines highlight the biological significance threshold of +/-2-fold change in spectral counts or intensity between DR versus AL, while the non-axial red dotted horizontal line with asterisk highlights the statistical significance threshold of *P* <0.05. Blue (AL enriched) and green (DR enriched) colored rows and dots indicate persulfidated proteins reaching both biological- and statistical- thresholds. Gray color rows and dots indicate persulfidated proteins not reaching the criteria for both biological and statistical significance under either diet.

File Name: **Supplementary Data 5_Acc Num Shared Persulfidated Prots WT Liv, Kid, Mus, Br**

Description: **Accession numbers for the shared persulfidated proteins in 6-month old CGL WT liver, kidney, muscle, and brain.** Listing of the 209 (spectral counting, tab 1) and 303 (intensity, tab 2) persulfidated proteins common in liver, kidney, muscle, and brain of 6-month old CGL WT mice. Additionally included is the Venn diagram visualizing the common proteins and their KEGG biological function and pathway enrichment via g:Profiler analysis. Statistical significance for pathway enrichment plotted as the adjusted –log_10_ (*P*-Value) and were auto-calculated via the g:Profiler g:SCS algorithm for KEGG database that utilizes multiple testing correction.

File Name: **Supplementary Data 6_Pathway Enrich WT LIVER**

Description: **Pathway enrichment with persulfidated proteins in 6-month old CGL WT Liver.** Information on the name of the pathway enriched, the adjusted *P*-values**,** and the found persulfidated proteins/genes involved in that pathway from spectral counting (tab 1) or MS1 intensity (tab 2). Statistical significance for pathway enrichment plotted as the adjusted –log_10_ (*P*-Value) and were auto-calculated via the g:Profiler g:SCS algorithm for KEGG database that utilizes multiple testing correction. Green headings are from proteins enriched under DR, blue heading are from protein enriched under AL, and gray headings are for proteins that did not meet both significance thresholds.

File Name: **Supplementary Data 7_Pathway Enrich WT KIDNEY**

Description: **Pathway enrichment with persulfidated proteins in 6-month old CGL WT Kidney.** Information on the name of the pathway enriched, the adjusted *P*-values**,** and the found persulfidated proteins/genes involved in that pathway from spectral counting (tab 1) or MS1 intensity (tab 2). Statistical significance for pathway enrichment plotted as the adjusted –log_10_ (*P*-Value) and were auto-calculated via the g:Profiler g:SCS algorithm for KEGG database that utilizes multiple testing correction. Green headings are from proteins enriched under DR, blue heading are from protein enriched under AL, and gray headings are for proteins that did not meet both significance thresholds.

File Name: **Supplementary Data 8_Pathway Enrich MUSCLE**

Description: **Pathway enrichment with persulfidated proteins in 6-month old CGL WT muscle.** Information on the name of the pathway enriched, the adjusted *P*-values**,** and the found persulfidated proteins/genes involved in that pathway from spectral counting (tab 1) or MS1 intensity (tab 2). Statistical significance for pathway enrichment plotted as the adjusted –log_10_ (*P*-Value) and were auto-calculated via the g:Profiler g:SCS algorithm for KEGG database that utilizes multiple testing correction. Green headings are from proteins enriched under DR, blue heading are from protein enriched under AL, and gray headings are for proteins that did not meet both significance thresholds.

File Name: **Supplementary Data 9_Pathway Enrich WT BRAIN**

Description: **Pathway enrichment with persulfidated proteins in 6-month old CGL WT brain.** Information on the name of the pathway enriched, the adjusted *P*-values**,** and the found persulfidated proteins/genes involved in that pathway from spectral counting (tab 1) or MS1 intensity (tab 2). Statistical significance for pathway enrichment plotted as the adjusted –log_10_ (*P*-Value) and were auto-calculated via the g:Profiler g:SCS algorithm for KEGG database that utilizes multiple testing correction. Green headings are from proteins enriched under DR, blue heading are from protein enriched under AL, and gray headings are for proteins that did not meet both significance thresholds.

File Name: **Supplementary Data 10_WT HEART Persulfidome**

Description: **6-month old CGL WT heart persulfidome.** Information on the tissue-specific persulfidome, including protein names, accession numbers, and the DR to AL ratio from spectral counting (tab 1) or MS1 intensity (tab 2). The *P*-value is calculated by comparing the individual spectral count or intensity values for each identified persulfidated protein in a specific tissue from AL versus DR fed mice via a 2-sided Student’s *t* test. AL; *n* = 4 mice/group versus DR; *n* = 5 mice/group .The accompanying volcano plot displays the differentially abundant persulfidated proteins. The log_2_(Fold Change DR:AL) X-axis displays the average fold change in spectral counts or intensity values for each identified persulfidated protein while the –log_10_ Y-axis displays the calculated *P*-value. The non-axial red dotted vertical lines highlight the biological significance threshold of +/-2-fold change in spectral counts or intensity between DR versus AL, while the non-axial red dotted horizontal line with asterisk highlights the statistical significance threshold of *P* <0.05. Blue (AL enriched) and green (DR enriched) colored rows and dots indicate persulfidated proteins reaching both biological- and statistical- thresholds. Gray color rows and dots indicate persulfidated proteins not reaching the criteria for both biological and statistical significance under either diet.

File Name: **Supplementary Data 11_WT PLASMA Persulfidome**

Description: **6-month old CGL WT plasma persulfidome.** Information on the tissue-specific persulfidome, including protein names, accession numbers, and the DR to AL ratio from spectral counting (tab 1) or MS1 intensity (tab 2). The *P*-value is calculated by comparing the individual spectral count or intensity values for each identified persulfidated protein in a specific tissue from AL versus DR fed mice via a 2-sided Student’s *t* test. AL; *n* = 4 mice/group versus DR; *n* = 5 mice/group .The accompanying volcano plot displays the differentially abundant persulfidated proteins. The log_2_(Fold Change DR:AL) X-axis displays the average fold change in spectral counts or intensity values for each identified persulfidated protein while the –log_10_ Y-axis displays the calculated *P*-value. The non-axial red dotted vertical lines highlight the biological significance threshold of +/-2-fold change in spectral counts or intensity between DR versus AL, while the non-axial red dotted horizontal line with asterisk highlights the statistical significance threshold of *P* <0.05. Blue (AL enriched) and green (DR enriched) colored rows and dots indicate persulfidated proteins reaching both biological- and statistical- thresholds. Gray color rows and dots indicate persulfidated proteins not reaching the criteria for both biological and statistical significance under either diet.

File Name: **Supplementary Data 12_Acc Num Shared Persulfidated Prots in WT Heart and Plasma**

Description: **Accession numbers for the shared persulfidated proteins in 6-month old CGL WT heart and plasma.** Listing of the 78 (spectral counting, tab 1) and 97 (intensity, tab 2) persulfidated proteins common heart and plasma of 6-month old CGL WT mice. Additionally included is the Venn diagram visualizing the common proteins and their KEGG biological function and pathway enrichment via g:Profiler analysis. Statistical significance for pathway enrichment plotted as the adjusted –log_10_ (*P*-Value) and were auto-calculated via the g:Profiler g:SCS algorithm for KEGG database that utilizes multiple testing correction.

File Name: **Supplementary Data 13_Pathway Enrich WT HEART**

Description: **Pathway enrichment with persulfidated proteins in 6-month old CGL WT heart.** Information on the name of the pathway enriched, the adjusted *P*-values**,** and the found persulfidated proteins/genes involved in that pathway from spectral counting (tab 1) or MS1 intensity (tab 2). Statistical significance for pathway enrichment plotted as the adjusted –log_10_ (*P*-Value) and were auto-calculated via the g:Profiler g:SCS algorithm for KEGG database that utilizes multiple testing correction. Green headings are from proteins enriched under DR, blue heading are from protein enriched under AL, and gray headings are for proteins that did not meet both significance thresholds.

File Name: **Supplementary Data 14_Pathway enrich WT PLASMA**

Description: **Pathway enrichment with persulfidated proteins in 6-month old CGL WT plasma.** Information on the name of the pathway enriched, the adjusted *P*-values**,** and the found persulfidated proteins/genes involved in that pathway from spectral counting (tab 1) or MS1 intensity (tab 2). Statistical significance for pathway enrichment plotted as the adjusted –log_10_ (*P*-Value) and were auto-calculated via the g:Profiler g:SCS algorithm for KEGG database that utilizes multiple testing correction. Green headings are from proteins enriched under DR, blue heading are from protein enriched under AL, and gray headings are for proteins that did not meet both significance thresholds.

File Name: **Supplementary Data 15_Acc Num Shared Persulfidated Prots in all 6 WT tissues**

Description: **Accession numbers for the shared persulfidated proteins in 6-month old CGL WT liver, kidney, muscle, brain, heart, and plasma.** Listing of the 28 (spectral counting, tab 1) and 32 (intensity, tab 2) persulfidated proteins common heart and plasma of 6-month old CGL WT mice. Additionally included is the Venn diagram visualizing the common proteins and their KEGG biological function and pathway enrichment via g:Profiler analysis. Statistical significance for pathway enrichment plotted as the adjusted –log_10_ (*P*-Value) and were auto-calculated via the g:Profiler g:SCS algorithm for KEGG database that utilizes multiple testing correction.

File Name: **Supplementary Data 16_Aged C57BL6 LIVER Persulfidome**

Description: **Aged C57BL/6 liver persulfidome.** Information on the tissue-specific persulfidome, including protein names, accession numbers, and the EOD fasting to AL ratio from spectral counting (tab 1) or MS1 intensity (tab 2). The *P*-value is calculated by comparing the individual spectral count or intensity values for each identified persulfidated protein in a specific tissue from AL versus EOD fasted mice via a 2-sided Student’s *t* test. AL; *n* = 5 mice/group versus EOD; *n* = 5 mice/group. The accompanying volcano plot displays the differentially abundant persulfidated proteins. The log_2_(Fold Change EOD:AL) X-axis displays the average fold change in spectral counts or intensity values for each identified persulfidated protein while the –log_10_ Y-axis displays the calculated *P*-value. The non-axial red dotted vertical lines highlight the biological significance threshold of +/-2-fold change in spectral counts or intensity between EOD versus AL, while the non-axial red dotted horizontal line with asterisk highlights the statistical significance threshold of *P* <0.05. Blue (AL enriched) and green (EOD enriched) colored rows and dots indicate persulfidated proteins reaching both biological- and statistical- thresholds. Gray color rows and dots indicate persulfidated proteins not reaching the criteria for both biological and statistical significance under either diet.

File Name: **Supplementary Data 17_Aged C57BL6 KIDNEY Persulfidome**

Description: **Aged C57BL/6 kidney persulfidome.** Information on the tissue-specific persulfidome, including protein names, accession numbers, and the EOD fasting to AL ratio from spectral counting (tab 1) or MS1 intensity (tab 2). The *P*-value is calculated by comparing the individual spectral count or intensity values for each identified persulfidated protein in a specific tissue from AL versus EOD fasted mice via a 2-sided Student’s *t* test. AL; *n* = 5 mice/group versus EOD; *n* = 5 mice/group. The accompanying volcano plot displays the differentially abundant persulfidated proteins. The log_2_(Fold Change EOD:AL) X-axis displays the average fold change in spectral counts or intensity values for each identified persulfidated protein while the –log_10_ Y-axis displays the calculated *P*-value. The non-axial red dotted vertical lines highlight the biological significance threshold of +/-2-fold change in spectral counts or intensity between EOD versus AL, while the non-axial red dotted horizontal line with asterisk highlights the statistical significance threshold of *P* <0.05. Blue (AL enriched) and green (EOD enriched) colored rows and dots indicate persulfidated proteins reaching both biological- and statistical- thresholds. Gray color rows and dots indicate persulfidated proteins not reaching the criteria for both biological and statistical significance under either diet.

File Name: **Supplementary Data 18_Aged C57BL6 MUSCLE Persulfidome**

Description: **Aged C57BL/6 muscle persulfidome.** Information on the tissue-specific persulfidome, including protein names, accession numbers, and the EOD fasting to AL ratio from spectral counting (tab 1) or MS1 intensity (tab 2). The *P*-value is calculated by comparing the individual spectral count or intensity values for each identified persulfidated protein in a specific tissue from AL versus EOD fasted mice via a 2-sided Student’s *t* test. AL; *n* = 5 mice/group versus EOD; *n* = 5 mice/group. The accompanying volcano plot displays the differentially abundant persulfidated proteins. The log_2_(Fold Change EOD:AL) X-axis displays the average fold change in spectral counts or intensity values for each identified persulfidated protein while the –log_10_ Y-axis displays the calculated *P*-value. The non-axial red dotted vertical lines highlight the biological significance threshold of +/-2-fold change in spectral counts or intensity between EOD versus AL, while the non-axial red dotted horizontal line with asterisk highlights the statistical significance threshold of *P* <0.05. Blue (AL enriched) and green (EOD enriched) colored rows and dots indicate persulfidated proteins reaching both biological- and statistical- thresholds. Gray color rows and dots indicate persulfidated proteins not reaching the criteria for both biological and statistical significance under either diet.

File Name: **Supplementary Data 19_Aged C57BL6 BRAIN Persulfidome**

Description: **Aged C57BL/6 brain persulfidome.** Information on the tissue-specific persulfidome, including protein names, accession numbers, and the EOD fasting to AL ratio from spectral counting (tab 1) or MS1 intensity (tab 2). The *P*-value is calculated by comparing the individual spectral count or intensity values for each identified persulfidated protein in a specific tissue from AL versus EOD fasted mice via a 2-sided Student’s *t* test. AL; *n* = 5 mice/group versus EOD; *n* = 5 mice/group. The accompanying volcano plot displays the differentially abundant persulfidated proteins. The log_2_(Fold Change EOD:AL) X-axis displays the average fold change in spectral counts or intensity values for each identified persulfidated protein while the –log_10_ Y-axis displays the calculated *P*-value. The non-axial red dotted vertical lines highlight the biological significance threshold of +/-2-fold change in spectral counts or intensity between EOD versus AL, while the non-axial red dotted horizontal line with asterisk highlights the statistical significance threshold of *P* <0.05. Blue (AL enriched) and green (EOD enriched) colored rows and dots indicate persulfidated proteins reaching both biological- and statistical- thresholds. Gray color rows and dots indicate persulfidated proteins not reaching the criteria for both biological and statistical significance under either diet.

File Name: **Supplementary Data 20_Aged C57BL6 HEART Persulfidome**

Description: **Aged C57BL/6 heart persulfidome.** Information on the tissue-specific persulfidome, including protein names, accession numbers, and the EOD fasting to AL ratio from spectral counting (tab 1) or MS1 intensity (tab 2). The *P*-value is calculated by comparing the individual spectral count or intensity values for each identified persulfidated protein in a specific tissue from AL versus EOD fasted mice via a 2-sided Student’s *t* test. AL; *n* = 5 mice/group versus EOD; *n* = 5 mice/group. The accompanying volcano plot displays the differentially abundant persulfidated proteins. The log_2_(Fold Change EOD:AL) X-axis displays the average fold change in spectral counts or intensity values for each identified persulfidated protein while the –log_10_ Y-axis displays the calculated *P*-value. The non-axial red dotted vertical lines highlight the biological significance threshold of +/-2-fold change in spectral counts or intensity between EOD versus AL, while the non-axial red dotted horizontal line with asterisk highlights the statistical significance threshold of *P* <0.05. Blue (AL enriched) and green (EOD enriched) colored rows and dots indicate persulfidated proteins reaching both biological- and statistical- thresholds. Gray color rows and dots indicate persulfidated proteins not reaching the criteria for both biological and statistical significance under either diet.

File Name: **Supplementary Data 21_CGL KO LIVER Persulfidome**

Description: **6-month old CGL KO liver persulfidome.** Information on the tissue-specific persulfidome, including protein names, accession numbers, and the DR to AL ratio from spectral counting (tab 1) or MS1 intensity (tab 2). The *P*-value is calculated by comparing the individual spectral count or intensity values for each identified persulfidated protein in a specific tissue from AL versus DR fed mice via a 2-sided Student’s *t* test. AL; *n* = 3 mice/group versus DR; *n* = 3 mice/group .The accompanying volcano plot displays the differentially abundant persulfidated proteins. The log_2_(Fold Change DR:AL) X-axis displays the average fold change in spectral counts or intensity values for each identified persulfidated protein while the –log_10_ Y-axis displays the calculated *P*-value. The non-axial red dotted vertical lines highlight the biological significance threshold of +/-2-fold change in spectral counts or intensity between DR versus AL, while the non-axial red dotted horizontal line with asterisk highlights the statistical significance threshold of *P* <0.05. Blue (AL enriched) and green (DR enriched) colored rows and dots indicate persulfidated proteins reaching both biological- and statistical- thresholds. Gray color rows and dots indicate persulfidated proteins not reaching the criteria for both biological and statistical significance under either diet.

File Name: **Supplementary Data 22_Acc Num CGL Dependent Persulfidated Proteins LIVER**

Description: **Accession numbers for the CGL dependent liver persulfidated proteins.** Listing of the 339 (spectral counting, tab 1) and 446 (intensity, tab 2) persulfidated liver proteins unique to 6-month old CGL WT mice. Additionally included is the Venn diagram visualizing the unique proteins and their KEGG biological function and pathway enrichment via g:Profiler analysis. Statistical significance for pathway enrichment plotted as the adjusted –log_10_ (*P*-Value) and were auto-calculated via the g:Profiler g:SCS algorithm for KEGG database that utilizes multiple testing correction.

File Name: **Supplementary Data 23_CGL KO KIDNEY Persulfidome**

Description: **6-month old CGL KO kidney persulfidome.** Information on the tissue-specific persulfidome, including protein names, accession numbers, and the DR to AL ratio from spectral counting (tab 1) or MS1 intensity (tab 2). The *P*-value is calculated by comparing the individual spectral count or intensity values for each identified persulfidated protein in a specific tissue from AL versus DR fed mice via a 2-sided Student’s *t* test. AL; *n* = 3 mice/group versus DR; *n* = 3 mice/group .The accompanying volcano plot displays the differentially abundant persulfidated proteins. The log_2_(Fold Change DR:AL) X-axis displays the average fold change in spectral counts or intensity values for each identified persulfidated protein while the –log_10_ Y-axis displays the calculated *P*-value. The non-axial red dotted vertical lines highlight the biological significance threshold of +/-2-fold change in spectral counts or intensity between DR versus AL, while the non-axial red dotted horizontal line with asterisk highlights the statistical significance threshold of *P* <0.05. Blue (AL enriched) and green (DR enriched) colored rows and dots indicate persulfidated proteins reaching both biological- and statistical- thresholds. Gray color rows and dots indicate persulfidated proteins not reaching the criteria for both biological and statistical significance under either diet.

File Name: **Supplementary Data 24_Acc Num CGL Dependent Persulfidated Proteins KIDNEY**

Description: **Accession numbers for the CGL dependent kidney persulfidated proteins.** Listing of the 333 (spectral counting, tab 1) and 413 (intensity, tab 2) persulfidated kidney proteins unique to 6-month old CGL WT mice. Additionally included is the Venn diagram visualizing the unique proteins and their KEGG biological function and pathway enrichment via g:Profiler analysis. Statistical significance for pathway enrichment plotted as the adjusted –log_10_ (*P*-Value) and were auto-calculated via the g:Profiler g:SCS algorithm for KEGG database that utilizes multiple testing correction.

File Name: **Supplementary Data 25_CGL KO MUSCLE Persulfidome**

Description: **6-month old CGL KO muscle persulfidome.** Information on the tissue-specific persulfidome, including protein names, accession numbers, and the DR to AL ratio from spectral counting (tab 1) or MS1 intensity (tab 2). The *P*-value is calculated by comparing the individual spectral count or intensity values for each identified persulfidated protein in a specific tissue from AL versus DR fed mice via a 2-sided Student’s *t* test. AL; *n* = 3 mice/group versus DR; *n* = 3 mice/group .The accompanying volcano plot displays the differentially abundant persulfidated proteins. The log_2_(Fold Change DR:AL) X-axis displays the average fold change in spectral counts or intensity values for each identified persulfidated protein while the –log_10_ Y-axis displays the calculated *P*-value. The non-axial red dotted vertical lines highlight the biological significance threshold of +/-2-fold change in spectral counts or intensity between DR versus AL, while the non-axial red dotted horizontal line with asterisk highlights the statistical significance threshold of *P* <0.05. Blue (AL enriched) and green (DR enriched) colored rows and dots indicate persulfidated proteins reaching both biological- and statistical- thresholds. Gray color rows and dots indicate persulfidated proteins not reaching the criteria for both biological and statistical significance under either diet.

File Name: **Supplementary Data 26_CGL KO BRAIN Persulfidome**

Description: **6-month old CGL KO brain persulfidome.** Information on the tissue-specific persulfidome, including protein names, accession numbers, and the DR to AL ratio from spectral counting (tab 1) or MS1 intensity (tab 2). The *P*-value is calculated by comparing the individual spectral count or intensity values for each identified persulfidated protein in a specific tissue from AL versus DR fed mice via a 2-sided Student’s *t* test. AL; *n* = 3 mice/group versus DR; *n* = 3 mice/group .The accompanying volcano plot displays the differentially abundant persulfidated proteins. The log_2_(Fold Change DR:AL) X-axis displays the average fold change in spectral counts or intensity values for each identified persulfidated protein while the –log_10_ Y-axis displays the calculated *P*-value. The non-axial red dotted vertical lines highlight the biological significance threshold of +/-2-fold change in spectral counts or intensity between DR versus AL, while the non-axial red dotted horizontal line with asterisk highlights the statistical significance threshold of *P* <0.05. Blue (AL enriched) and green (DR enriched) colored rows and dots indicate persulfidated proteins reaching both biological- and statistical- thresholds. Gray color rows and dots indicate persulfidated proteins not reaching the criteria for both biological and statistical significance under either diet.

File Name: **Supplementary Data 27_CGL KO HEART Persulfidome**

Description: **6-month old CGL KO heart persulfidome.** Information on the tissue-specific persulfidome, including protein names, accession numbers, and the DR to AL ratio from spectral counting (tab 1) or MS1 intensity (tab 2). The *P*-value is calculated by comparing the individual spectral count or intensity values for each identified persulfidated protein in a specific tissue from AL versus DR fed mice via a 2-sided Student’s *t* test. AL; *n* = 3 mice/group versus DR; *n* = 3 mice/group .The accompanying volcano plot displays the differentially abundant persulfidated proteins. The log_2_(Fold Change DR:AL) X-axis displays the average fold change in spectral counts or intensity values for each identified persulfidated protein while the –log_10_ Y-axis displays the calculated *P*-value. The non-axial red dotted vertical lines highlight the biological significance threshold of +/-2-fold change in spectral counts or intensity between DR versus AL, while the non-axial red dotted horizontal line with asterisk highlights the statistical significance threshold of *P* <0.05. Blue (AL enriched) and green (DR enriched) colored rows and dots indicate persulfidated proteins reaching both biological- and statistical- thresholds. Gray color rows and dots indicate persulfidated proteins not reaching the criteria for both biological and statistical significance under either diet.

File Name: **Supplementary Data 28_Acc Num CGL Dependent Persulfidated Proteins MUSCLE**

Description: **Accession numbers for the CGL dependent muscle persulfidated proteins.** Listing of the 284 (spectral counting, tab 1) and 309 (intensity, tab 2) persulfidated muscle proteins unique to 6-month old CGL WT mice. Additionally included is the Venn diagram visualizing the unique proteins.

File Name: **Supplementary Data 29_Acc Num CGL Dependnet Persulfidated Proteins BRAIN**

Description: **Accession numbers for the CGL dependent brain persulfidated proteins.** Listing of the 459 (spectral counting, tab 1) and 580 (intensity, tab 2) persulfidated brain proteins unique to 6-month old CGL WT mice. Additionally included is the Venn diagram visualizing the unique proteins.

File Name: **Supplementary Data 30_Acc Num CGL Dependent Persulfidated Proteins HEART**

Description: **Accession numbers for the CGL dependent heart persulfidated proteins.** Listing of the 292 (spectral counting, tab 1) and 358 (intensity, tab 2) persulfidated heart proteins unique to 6-month old CGL WT mice. Additionally included is the Venn diagram visualizing the unique proteins.

File Name: **Supplementary Data 31_Gray Dot Persulfidated Protein Enrich Analysis from CGL WT KO Tissues**

Description: **Gray dot persulfidated protein enrichment analysis from 5 CGL WT and KO tissues.** Numbers of persulfidated proteins not reaching both significance thresholds but being skewed toward enrichment under DR or AL in 6-month old CGL WT and KO mice as determined by spectral counting (tab 1) or MS1 intensity (tab 2).

File Name: **Supplementary Data 32_1 year old CGL WT vs KO KIDNEY Prot Level BTA**

Description: **1-year old 50% DR CGL WT and KO kidney persulfidome utilizing protein level BTA.** Information on the tissue-specific persulfidome, including protein names, accession numbers, and the WT to KO ratio from MS1 intensity. The *P*-value is calculated by comparing the individual intensity values for each identified persulfidated protein in a specific tissue from WT versus KO mice both fed 1-week 50% DR via a 2-sided Student’s *t* test. WT; *n* = 3mice/group versus KO; *n* = 3 mice/group .The accompanying volcano plot displays the differentially abundant persulfidated proteins. The log_2_(Fold Change WT:KO) X-axis displays the average fold change in intensity values for each identified persulfidated protein while the –log_10_ Y-axis displays the calculated *P*-value. The non-axial red dotted vertical lines highlight the biological significance threshold of +/-2-fold change in spectral counts or intensity between WT versus KO, while the non-axial red dotted horizontal line with asterisk highlights the statistical significance threshold of *P* <0.05. Blue (KO enriched) and green (WT enriched) colored rows and dots indicate persulfidated proteins reaching both biological- and statistical- thresholds. Gray color rows and dots indicate persulfidated proteins not reaching the criteria for both biological and statistical significance.

File Name: **Supplementary Data 33_1 year old CGL WT vs KO BRAIN Prot Level BTA**

Description: **1-year old 50% DR CGL WT and KO brain persulfidome utilizing protein level BTA.** Information on the tissue-specific persulfidome, including protein names, accession numbers, and the WT to KO ratio from MS1 intensity. The *P*-value is calculated by comparing the individual intensity values for each identified persulfidated protein in a specific tissue from WT versus KO mice both fed 1-week 50% DR via a 2-sided Student’s *t* test. WT; *n* = 3mice/group versus KO; *n* = 3 mice/group .The accompanying volcano plot displays the differentially abundant persulfidated proteins. The log_2_(Fold Change WT:KO) X-axis displays the average fold change in intensity values for each identified persulfidated protein while the –log_10_ Y-axis displays the calculated *P*-value. The non-axial red dotted vertical lines highlight the biological significance threshold of +/-2-fold change in spectral counts or intensity between WT versus KO, while the non-axial red dotted horizontal line with asterisk highlights the statistical significance threshold of *P* <0.05. Blue (KO enriched) and green (WT enriched) colored rows and dots indicate persulfidated proteins reaching both biological- and statistical- thresholds. Gray color rows and dots indicate persulfidated proteins not reaching the criteria for both biological and statistical significance.

File Name: **Supplementary Data 34_1 year old CGL WT vs KO KIDNEY Peptide Level BTA**

Description: **1-year old 50% CGL WT and KO kidney persulfidome utilizing peptide level BTA.** Information on the tissue-specific persulfidome, including protein names, accession numbers, and the WT to KO ratio from MS1 intensity. The *P*-value is calculated by comparing the individual intensity values for each identified persulfidated protein in a specific tissue from WT versus KO mice both fed 1-week 50% DR via a 2-sided Student’s *t* test. WT; *n* = 3mice/group versus KO; *n* = 3 mice/group .The accompanying volcano plot displays the differentially abundant persulfidated proteins. The log_2_(Fold Change WT:KO) X-axis displays the average fold change in intensity values for each identified persulfidated protein while the –log_10_ Y-axis displays the calculated *P*-value. The non-axial red dotted vertical lines highlight the biological significance threshold of +/-2-fold change in spectral counts or intensity between WT versus KO, while the non-axial red dotted horizontal line with asterisk highlights the statistical significance threshold of *P* <0.05. Blue (KO enriched) and green (WT enriched) colored rows and dots indicate persulfidated proteins reaching both biological- and statistical- thresholds. Gray color rows and dots indicate persulfidated proteins not reaching the criteria for both biological and statistical significance.

File Name: **Supplemenatry Data 35_1 year old CGL WT vs KO BRAIN Peptide Level BTA**

Description: **1-year old 50% DR CGL WT and KO brain persulfidome utilizing peptide level BTA.** Information on the tissue-specific persulfidome, including protein names, accession numbers, and the WT to KO ratio from MS1 intensity. The *P*-value is calculated by comparing the individual intensity values for each identified persulfidated protein in a specific tissue from WT versus KO mice both fed 1-week 50% DR via a 2-sided Student’s *t* test. WT; *n* = 3mice/group versus KO; *n* = 3 mice/group .The accompanying volcano plot displays the differentially abundant persulfidated proteins. The log_2_(Fold Change WT:KO) X-axis displays the average fold change in intensity values for each identified persulfidated protein while the –log_10_ Y-axis displays the calculated *P*-value. The non-axial red dotted vertical lines highlight the biological significance threshold of +/-2-fold change in spectral counts or intensity between WT versus KO, while the non-axial red dotted horizontal line with asterisk highlights the statistical significance threshold of *P* <0.05. Blue (KO enriched) and green (WT enriched) colored rows and dots indicate persulfidated proteins reaching both biological- and statistical- thresholds. Gray color rows and dots indicate persulfidated proteins not reaching the criteria for both biological and statistical significance.

File Name: **Supplementary Data 36_1 year old 50% DR CGL WT vs KO Kidney Peptide level BTA iodoTMT**

Description: **1-year old 50% DR CGL WT and KO kidney persulfidome utilizing iodoTMT-labiled peptide level BTA.** (Tab 1) Information on the tissue-specific persulfidome, including protein names, accession numbers, and the WT to KO ratio from iodoTMT label intensity. The *P*-value is calculated by comparing the individual intensity values for each identified persulfidated protein in a specific tissue from WT versus KO mice both fed 1-week 50% DR via a 2-sided Student’s *t* test. WT; *n* = 3mice/group versus KO; *n* = 3 mice/group .The accompanying volcano plot displays the differentially abundant persulfidated proteins. The log_2_(Fold Change WT:KO) X-axis displays the average fold change in intensity values for each identified persulfidated protein while the –log_10_ Y-axis displays the calculated *P*-value. The non-axial red dotted vertical lines highlight the biological significance threshold of +/-2-fold change in spectral counts or intensity between WT versus KO, while the non-axial red dotted horizontal line with asterisk highlights the statistical significance threshold of *P* <0.05. Blue (KO enriched) and green (WT enriched) colored rows and dots indicate persulfidated proteins reaching both biological- and statistical- thresholds. Gray color rows and dots indicate persulfidated proteins not reaching the criteria for both biological and statistical significance. (Tab 2) Annotated peptide sequence and protein identification information for cysteine modifications identified through iodoTMT labeling.

File Name: **Supplementary Data 37_1 year old 50% DR CGL WT vs KO Brain Peptide level BTA iodoTMT**

Description: **1-year old 50% CGL WT and KO brain persulfidome utilizing iodoTMT-labiled peptide level BTA.** (Tab 1) Information on the tissue-specific persulfidome, including protein names, accession numbers, and the WT to KO ratio from iodoTMT label intensity. The *P*-value is calculated by comparing the individual intensity values for each identified persulfidated protein in a specific tissue from WT versus KO mice both fed 1-week 50% DR via a 2-sided Student’s *t* test. WT; *n* = 3mice/group versus KO; *n* = 3 mice/group .The accompanying volcano plot displays the differentially abundant persulfidated proteins. The log_2_(Fold Change WT:KO) X-axis displays the average fold change in intensity values for each identified persulfidated protein while the –log_10_ Y-axis displays the calculated *P*-value. The non-axial red dotted vertical lines highlight the biological significance threshold of +/-2-fold change in spectral counts or intensity between WT versus KO, while the non-axial red dotted horizontal line with asterisk highlights the statistical significance threshold of *P* <0.05. Blue (KO enriched) and green (WT enriched) colored rows and dots indicate persulfidated proteins reaching both biological- and statistical- thresholds. Gray color rows and dots indicate persulfidated proteins not reaching the criteria for both biological and statistical significance. (Tab 2) Annotated peptide sequence and protein identification information for cysteine modifications identified through iodoTMT labeling.

File Name: **Supplementary Data 38_6 month old AL fed CGL WT vs KO Kidney Peptide level BTA iodoTMT**

Description: **6-month old AL fed CGL WT and KO kidney persulfidome utilizing iodoTMT-labiled peptide level BTA.** (Tab 1) Information on the tissue-specific persulfidome, including protein names, accession numbers, and the WT to KO ratio from iodoTMT label intensity. The *P*-value is calculated by comparing the individual intensity values for each identified persulfidated protein in a specific tissue from WT versus KO mice both fed AL via a 2-sided Student’s *t* test. WT; *n* = 3mice/group versus KO; *n* = 3 mice/group .The accompanying volcano plot displays the differentially abundant persulfidated proteins. The log_2_(Fold Change WT:KO) X-axis displays the average fold change in intensity values for each identified persulfidated protein while the –log_10_ Y-axis displays the calculated *P*-value. The non-axial red dotted vertical lines highlight the biological significance threshold of +/-2-fold change in spectral counts or intensity between WT versus KO, while the non-axial red dotted horizontal line with asterisk highlights the statistical significance threshold of *P* <0.05. Blue (KO enriched) and green (WT enriched) colored rows and dots indicate persulfidated proteins reaching both biological- and statistical- thresholds. Gray color rows and dots indicate persulfidated proteins not reaching the criteria for both biological and statistical significance. (Tab 2) Annotated peptide sequence and protein identification information for cysteine modifications identified through iodoTMT labeling.

File Name: **Supplementary Data 39_Thermomixture Quality Control (QC1) Spectral Counting**

Description: **Mass Spec QC1 instrument quality control data information. (**Tab 1) File names, file information, and Figshare link locations for raw quality control (QC) 1 data. (Tab 2) Retention times for Thermomixture Quality Control standards corresponding to time periods experimental samples were analyzed via mass spectrometry.
