# Supplementary material for: Dietary restriction transforms the mammalian protein persulfidome in a tissue-specific and cystathionine γ-lyase-dependent manner
Source: Nat Commun. 2021 Mar 19;12:1745. doi: 10.1038/s41467-021-22001-w (PMC7979915; doi:10.1038/s41467-021-22001-w)
Supplement: Supplementary file 43 — Reporting Summary [file 41467_2021_22001_MOESM43_ESM.pdf]

nature research

Corresponding author(s): Christopher Hine

Last updated by author(s): Feb 2, 2021

Reporting Summary

Nature Research wishes to improve the reproducibility of the work that we publish. This form provides structure for consistency and transparency in reporting. For further information on Nature Research policies, see our Editorial Policies and the Editorial Policy Checklist.

Statistics

For all statistical analyses, confirm that the following items are present in the figure legend, table legend, main text, or Methods section.

n/a	Confirmed	
☐	☒	The exact sample size (*n*) for each experimental group/condition, given as a discrete number and unit of measurement
☐	☒	A statement on whether measurements were taken from distinct samples or whether the same sample was measured repeatedly
☐	☒	The statistical test(s) used AND whether they are one- or two-sided<br>*Only common tests should be described solely by name; describe more complex techniques in the Methods section.*
☐	☒	A description of all covariates tested
☐	☒	A description of any assumptions or corrections, such as tests of normality and adjustment for multiple comparisons
☐	☒	A full description of the statistical parameters including central tendency (e.g. means) or other basic estimates (e.g. regression coefficient) AND variation (e.g. standard deviation) or associated estimates of uncertainty (e.g. confidence intervals)
☐	☒	For null hypothesis testing, the test statistic (e.g. *F*, *t*, *r*) with confidence intervals, effect sizes, degrees of freedom and *P* value noted<br>*Give P values as exact values whenever suitable.*
☒	☐	For Bayesian analysis, information on the choice of priors and Markov chain Monte Carlo settings
☐	☒	For hierarchical and complex designs, identification of the appropriate level for tests and full reporting of outcomes
☒	☐	Estimates of effect sizes (e.g. Cohen's *d*, Pearson's *r*), indicating how they were calculated

Our web collection on statistics for biologists contains articles on many of the points above.

Software and code

Policy information about availability of computer code

Data collection	Data on peptides were collected on a ThermoFisher Scientific UltiMate 3000 HPLC system (ThermoFisher Scientific, Bremen, Germany) interfaced with a ThermoFisher Scientific Orbitrap Fusion Lumos Tribrid mass spectrometer (Thermo Scientific, Bremen, Germany).<br><br>Images of protein gels and lead acetate/lead sulfide filter papers were collected on an Epson Perfection V500 scanner run with Epson software version 3.7.7.3US.<br><br>Western blot data was collected on an Amersham Imager 600 (General Electric)
Data analysis	ImageJ 1.51n IntDen function was used to analyze digital images of lead acetate/lead sulfide papers for H2S production capacity.<br><br>Mascot, SEQUEST and Scaffold software packages were used for label-free quantitative and qualitative proteomics analysis. These software were used for protein identification by converting raw spectrometric data into protein IDs and for relative quantification of protein abundance via label-free spectral counting. For protein identification, three search engines were used including Mascot bundled with Discoverer Daemon 1.4, Sequest which is bundled into Proteome Discoverer 1.4, and X!Tandem which is bundled into Scaffold 4.8.7. For the Mascot searches, primary raw MS/MS data were converted into its MGF (Mascot Generic File) format files by using Discoverer Daemon 1.4 software (Licensed under Cleveland Clinic Proteomics Core). Scaffold software (version Scaffold_4.8.7, Proteome Software Inc, Portland, OR) was used to validate peptide and protein identifications and create comprehensive lists of target proteins.<br><br>We also used MaxQuant version 1.6.14.0 wtih Perseus version 1.6.12.0 for further analysis and validation of raw data files for label free MS1 intensity-based quantitation<br><br>MS raw data files of iodoTMT labeled peptides were analyzed by Proteome Discoverer version 2.4.1.15 (Thermo Fisher Scientific).

The online-based web server g:Profiler [https://biit.cs.ut.ee/gprofiler/gost] was used for functional enrichment analysis of identified persulfidated proteins. Gene ID's or accession number of enriched proteins were used in the g:GOSt (Gene Group Functional Profiling) identifier tool with the built in g:SCS algorithm [https://biit.cs.ut.ee/gprofiler/page/docs] to detect biological pathways significantly enriched from the KEGG database.

Statistical significance and data display were generated in Microsoft Excel (from Office 2013), GraphPad Prism (version 5), Origin (version 2018) [https://www.originlab.com/], and Venny 2.1.0 software [https://bioinfogp.cnb.csic.es/tools/venny/].

For manuscripts utilizing custom algorithms or software that are central to the research but not yet described in published literature, software must be made available to editors and reviewers. We strongly encourage code deposition in a community repository (e.g. GitHub). See the Nature Research guidelines for submitting code & software for further information.

Data

Policy information about availability of data

All manuscripts must include a data availability statement. This statement should provide the following information, where applicable:

- Accession codes, unique identifiers, or web links for publicly available datasets
- A list of figures that have associated raw data
- A description of any restrictions on data availability

Data Availability Statement: The authors declare the data supporting the findings of this study are available within the paper and the Supplemental Information file (containing Supplementary Figures 1-9) and in the Supplementary Data files 1-40. Source image data and graphical values are available in Supplementary Data file 39. Furthermore, raw proteomics-related data have been uploaded to the ProteomeXchange Consortium via the PRIDE partner repository with the following Dataset identifier information: Project accession PXD022888 with Project Webpage [http://www.ebi.ac.uk/pride/archive/projects/PXD022888] (from Proteome Discoverer 2.4 based analysis), Project accession PXD022956 with Project Webpage [http://www.ebi.ac.uk/pride/archive/projects/PXD022956] (from MaxQuant 1.6.14.0 based analysis), and Project accession PXD022954 with Project Webpage [http://www.ebi.ac.uk/pride/archive/projects/PXD022954] (from Scaffold 4.8.7 based analysis). Proteomic instrumental quality control data (QC1) available at Figshare: [https://figshare.com/s/941a9d9bc9cd39b0dac6] and [https://figshare.com/s/f613f80460bce4eb722d] and are summarized in Supplementary Data 40. RNA expression graphs of H2S producing and consuming proteins were generated utilizing the Mouse ENCODE project 55 with values extracted from the publically availably NCBI Mouse Gene Database at the following NCBI webpages: CGL: [https://www.ncbi.nlm.nih.gov/gene/107869]; CBS: [https://www.ncbi.nlm.nih.gov/gene/12411]; 3-MST: [https://www.ncbi.nlm.nih.gov/gene/246221]; and SQR [https://www.ncbi.nlm.nih.gov/gene/59010]. SwissProt/UniProt data used in mass spectrometry analysis software tools is from the publically available UniProt database [https://www.uniprot.org/]. Questions and requests for resources and data should be directed to and will be fulfilled by the Lead Contact, Christopher Hine (hinec@ccf.org).

Field-specific reporting

Please select the one below that is the best fit for your research. If you are not sure, read the appropriate sections before making your selection.

☒ Life sciences ☐ Behavioural & social sciences ☐ Ecological, evolutionary & environmental sciences

For a reference copy of the document with all sections, see nature.com/documents/nr-reporting-summary-flat.pdf

Life sciences study design

All studies must disclose on these points even when the disclosure is negative.

Sample size	Determination of sample sizes were not established using pre-experimental calculations, but were instead established empirically based on preliminary experiments showing strong differences between groups and genotypes so that running at minimum N=3-5 per group was sufficient to reach statistical significance for the final experiments. Previous work utilizing N = 3-5 per group that detected significant results regarding the impact of diet or CGL status on various physiological and redox endpoints can be found in Hine, et al. Cell 2015 [https://pubmed.ncbi.nlm.nih.gov/25542313/], Hine, et al. Cell Metabolism 2017 [https://pubmed.ncbi.nlm.nih.gov/28591635/], and Zivanovic, et al. Cell Metabolism [https://pubmed.ncbi.nlm.nih.gov/31735592/]. Sample sizes are provided throughout the Figure Legends as well as in the Materials and Methods section.
Data exclusions	No animals or samples were excluded from analysis. However, data on proteins not containing cysteine residues were removed as they were considered as definitive false positives. This information is described in Methods, Bioinformatics for peptide identification and quantification subsection, paragraph 2, and states: "Additionally, to remove any false positive hits, we individually analyzed all of the proteins identified to ensure each one contained at least one cysteine residue by using the mouse protein amino acid sequences from UniProt Knowledgebase protein database (Proteome_ID/Tax_ID: UP000000589/10090). Those that did not contain at least one cysteine residue, and thus could not theoretically be persulfidated (approx. ~ 2-4% of our initial findings), were removed from our database and from further analysis".
Replication	All measurements and data in this study were taken from distinct individual mice/biological samples and not the same sample measured repeatedly. N-values for the number of individual mice used are as follows (unless noted in the respective figure legends): 1) 6-month old CGL WT and KO mice: 4 mice for WT AL, 5 mice for WT DR, 3 mice for KO AL, and 3 mice for KO DR; 2) 12-month old CGL WT and KO mice: 3 mice for WT DR, and 3 mice for KO DR; and 3) 20-month C57BL/6 mice: 5 mice for AL, and 5 mice for EOD fasting. Thus, the experiments were performed in three different ages of mice and on different genetic backgrounds to provide rigor and reproducibility to our study. In repeating similar experiments in these different aged and genetic background mice with variations in the diet, we were successful at achieving similar results between experiments. We also did parallel analysis of the label-free mass spec datasets using spectral counting and MS1 intensity based approaches, and in both approaches achieved similar results, as can bee seen in Figure 8I.
Randomization	Details on animal randomization are found in: Methods, Animal husbandry and diet intervention subsection, paragraph 1: "Ad libitum food

	intake per cage was measured daily for up to four days to determine the correct amount to restrict to achieve 50% reduction in food intake. After randomly assigning ad libitum (AL) (n = 3-4/genotype/experiment) or diet restriction (DR) (n = 3-5/genotype/experiment) feeding to the cages, food intake and body mass were measured over the 1-week intervention." and in paragraph 2: "At approximately 20 months of age, cages were randomly assigned to either EOD fasting (n = 5) or AL access (n = 5) to the standard rodent chow."
Blinding	Times researchers were blinded and/or not involved in certain aspects of the work, this is described in Methods, Animal Husbandry and Diet Intervention subsection, paragraph 3. Primarily, researchers involved in the proteomics and mass spectrometry experiments were not involved in the live animal studies portion of this study, and likewise those involved in the live animal studies were not involved in the proteomics and mass spectrometry experiments. As described in the Randomization section above, mice were handled and randomly divided into control and dietary intervention groups in a manner to prevent bias based on mouse physiology, appearance, or weight. Subsequent analyses of tissues from these animals via proteomics techniques were completed by researchers initially blinded in regards to individual animal identify during initial processing of the tissues. However, the need for blinding was removed at the start of the biotin thiol assay and downstream proteomics work and analysis due to researcher expertise, availability, and the need to orient the precious and limited samples in the correct order for gel images and mass spectrometry.

Reporting for specific materials, systems and methods

We require information from authors about some types of materials, experimental systems and methods used in many studies. Here, indicate whether each material, system or method listed is relevant to your study. If you are not sure if a list item applies to your research, read the appropriate section before selecting a response.

Materials & experimental systems

n/a	Involved in the study
☐	☒ Antibodies
☒	☐ Eukaryotic cell lines
☒	☐ Palaeontology and archaeology
☐	☒ Animals and other organisms
☒	☐ Human research participants
☒	☐ Clinical data
☒	☐ Dual use research of concern

Methods

n/a	Involved in the study
☒	☐ ChIP-seq
☒	☐ Flow cytometry
☒	☐ MRI-based neuroimaging

Antibodies

Antibodies used	Only commercially available antibodies were used: 1) Anti-GAPDH antibody [6C5] (Abcam #ab8245) diluted 1:1,000 in 5% milk, 2) horeseradish peroxidase-conjugated goat anti-mouse secondary antibody (Invitrogen #62-6520) diluted 1:5,000 in 5% milk, 3) Anti-alpha Tubulin antibody (Abcam #ab4074) diluted 1:1,000 in 5% milk, and 4) horseradish peroxidase-conjugated goat anti-rabbit IgG secondary antibody (Abcam #97051) diluted 1:5,000 in 5% milk.
Validation	1,840 publications have references for the GAPDH antibody as per Abcam's webpage: https://www.abcam.com/gapdh-antibody-6c5-loading-control-ab8245.html?productWallTab=ShowAll. 226 publications have references for the alpha Tubulin antibody as per Abcam's webpage: https://www.abcam.com/alpha-tubulin-antibody-loading-control-ab4074-references.html#top-1048. 378 publications have references for Goat Anti-Rabbit IgG H&L (HRP) (ab97051): https://www.abcam.com/goat-rabbit-igg-hl-hrp-ab97051.html. 64 references are listed for Goat anti-Mouse IgG (H+L) Secondary Antibody, HRP (Invitrogen 62-6520): https://www.thermofisher.com/antibody/product/Goat-anti-Mouse-IgG-H-L-Secondary-Antibody-Polyclonal/62-6520

Animals and other organisms

Policy information about studies involving animals; ARRIVE guidelines recommended for reporting animal research

Laboratory animals	All experiments were performed with approval of the Cleveland Clinic Institutional Animal Care and Use Committee, protocol # 2016-1778, and followed the National Institutes of Health Guide for the Care and Use of Laboratory Animals. In experiments utilizing male cystathionine gamma-lyase (CGL) wildtype (WT) and knockout (KO) mice, the mice were bred and weaned at 21-23 days of age and maintained under standard barrier housing in the Cleveland Clinic Lerner Research Institute on a 14-hr light/10-hr dark cycle dark cycle, temperature between 20–23°C, 30%–70% relative humidity, and with initial ad libitum access to standard rodent food (Envigo #2918) and drinking water until the dietary intervention. Experimental cystathionine γ-lyase (CGL) mice were obtained from initial parental CGL Het x Het breeding to generate CGL WT and KO F1s, and then breeding CGL WT x WT or CGL KO x KO F1s to generate the littermate and/or age-matched F2 experimental animals. Mice were group housed with 3-5 mice per cage. The CGL WT and KO mice were originally generated on a mixed 129/C57BL/6 background and then subsequently rederived into pathogen free C57BL/6 mice at Jackson Laboratories prior to colony establishment at Cleveland Clinic. When mice were 6-months or 12-months old they were switched to the experimental AIN-93G-based diet (Research Diets D10012G-2V-Formula 1) and exposed to ad libitum access for several days to adapt to the new food and for monitoring intake. The experimental diet consists of 20% of calories from protein (casein-based containing approximately 2.9% methionine and 0.4% cysteine, with additional L-cystine supplemented to a final 1.5 g/1,000 g final diet composition by Research Diets, Inc.), 64% of calories from carbohydrate, and 16% calories from fat, and importantly has 2x concentrations of mineral mix S10022G, vitamin mix V10037, and choline bitartrate to avoid potential micronutrient malnutrition during 50% dietary restriction. The powdered food mix was added in a 1:1 ratio (gram:mL) to a 2% agar (Sigma #A1296) solution in water before solidification to a semi-solid consistency that lessens the potential for food hoarding in group housing and increases accuracy of food consumption measurement. Ad libitum food intake per cage was measured daily for up to four days to determine the correct amount to restrict to achieve 50% reduction in food intake. After randomly assigning ad libitum (AL) (n = 3-4/genotype/experiment) or diet restriction (DR) (n = 3-5/genotype/experiment) feeding to the cages, food intake and body

mass were measured over the 1-week intervention. AL fed mice were provided 24-hour access to the diet and the DR mice fed their calculated allotment near the start of their dark phase at 7pm to limit disturbances in circadian rhythms and feeding patterns between the two groups.

For aged mice experiments testing every-other-day (EOD) fasting, male C57BL/6 mice were obtained between 60-65 weeks of age (Stock No: 000664, Jackson Laboratories, Bar Harbor, ME) and group-housed (up to 5 same-sex mice per cage) in the Cleveland Clinic Lerner Research Institute Biological Resource Unit. The mice had ad libitum (AL) access to standard rodent chow (18.6% protein, 44.2% carbohydrate, and 6.2% fat; Teklad Global Rodent Diet #2918, Envigo, Madison, WI). At approximately 20 months of age, cages were randomly assigned to either EOD fasting (n = 5) or AL access (n = 5) to the standard rodent chow. The EOD fasting regimen consisted of repeated cycles of 24-hr consecutive removal of food access with water always available (fast day) followed by 24-hr access to food and water (fed day). The EOD fasting intervention proceeded for 2.5 months. To circumvent possible disturbances in circadian rhythms and feeding patterns in the Chow EOD group, the food was provided to or removed from the Chow EOD group just prior to the dark cycle onset at 7pm.

After the noted dietary intervention periods, mice were euthanized in the late morning (between 9am-12pm) via isoflurane anesthesia overdose followed by cervical dislocation prior to collecting liver, kidney, heart, muscle (quadriceps), brain, and plasma. Plasma was collected via retro-orbital bleed and the blood immediately placed into lithium-heparin-coated tubes (Terumo #T-MLH). Tubes were centrifuged to separate RBCs from the plasma. Collected tissues were immediately placed in 1.5 mL centrifuge tubes, flash frozen in liquid nitrogen, and then stored in the dark at -80oC until further analysis. Subsequent analysis of tissues was by researchers not involved in the live animal experiments and was initially done blinded in regards to individual animal identify but unblinded for downstream assays and data analysis.

Wild animals	This study did not use wild animals
Field-collected samples	This study did not use field-collected samples
Ethics oversight	All experiments were performed with approval of the Cleveland Clinic Institutional Animal Care and Use Committee, protocol # 2016-1778, and followed the National Institutes of Health Guide for the Care and Use of Laboratory Animals.

Note that full information on the approval of the study protocol must also be provided in the manuscript.
